# Association of different domains of physical activity with diabetic kidney disease: a population-based study

**DOI:** 10.3389/fendo.2024.1364028

**Published:** 2024-05-28

**Authors:** Pengfei He, Yuanyuan Deng, Shaoning Dong, Hongdian Li, Cong Liu, Yu Ma, Cheng Tang, Mianzhi Zhang

**Affiliations:** ^1^ Department of Nuphrology, Dongfang Hospital, Beijing University of Chinese Medicine, Beijing, China; ^2^ Department of Nephrology, Tianjin Academy of Traditional Chinese Medicine Affiliated Hospital, Tianjin, China; ^3^ Department of Endocrinology, Dongzhimen Hospital, Beijing University of Chinese Medicine, Beijing, China

**Keywords:** National health and nutrition examination survey (NHANES), domain-specific physical activity, diabetes, diabetic kidney disease, kidney function

## Abstract

**Background:**

The aim of this cross-sectional study was to elucidate the associations between various domains of physical activity, such as occupation-related (OPA), transportation-related (TPA), leisure-time (LTPA) and overall physical activity (PA), and diabetic kidney disease.

**Methods:**

Our study encompassed 2,633 participants, drawn from the cross-sectional surveys of the National Health and Nutrition Examination Survey (NHANES) between 2007 and 2018, and employed survey-weighted logistic regression, generalized linear regression, and restricted cubic spline (RCS) analyses to ascertain the relationship between different domains of physical activity and diabetic kidney disease.

**Results:**

After controlling for all confounders, multivariate logistic regression analyses revealed a lack of correlation between the various domains of physical activity and the prevalence of diabetic kidney disease. Multiple generalized linear regression analyses showed that durations of PA (β = 0.05, 95% CI, 0.01–0.09, P = 0.012) and TPA (β = 0.32, 95% CI, 0.10–0.55, P = 0.006) were positively associated with eGFR levels; and LTPA durations were inversely associated with UACR levels (β = -5.97, 95% CI, -10.50 - -1.44, P = 0.011). The RCS curves demonstrated a nonlinear relationship between PA, OPA, and eGFR, as well as a nonlinear correlation between PA and ACR. Subgroup and sensitivity analyses largely aligned with the outcomes of the multivariate generalized linear regression, underscoring the robustness of our findings.

**Conclusion:**

Our population-based study explored the association between different domains of physical activity and diabetic kidney disease. Contrary to our expectations, we found no significant association between the duration of physical activity across all domains and the prevalence of diabetic nephropathy. Nonetheless, renal function markers, including eGFR and UACR, exhibited significant correlations with the duration of total physical activity (TPA) and leisure-time physical activity (LTPA), respectively, among diabetic patients. Interestingly, our findings suggest that diabetic patients engage in physical activity to preserve renal function, ensuring moderate exercise durations not exceeding 35 hours per week.

## 1 Introduction

Diabetic kidney disease represents one of the most severe complications of diabetes mellitus, leading to kidney damage through mechanisms such as high plasma glucose-induced glomerular hyperfiltration and hypertension-induced alterations in renal hemodynamics. Notably, approximately one-third of individuals with diabetes exhibit renal function abnormalities (1) (2). According to recent statistics, the global adult population with diabetes has reached 537 million, and the prevalence of the disease is projected to surpass 780 million by 2045 (3). This trend suggests a likely increase in the prevalence of diabetic kidney disease, posing a significant public health challenge. Diabetic kidney disease is a principal cause of kidney failure and end-stage renal disease, profoundly impacting patients’ quality of life and elevating the risk of adverse outcomes, including cardiovascular disease, infections, and mortality (4). At present, the progression of diabetic kidney disease is primarily mitigated or delayed through lifestyle improvements such as diet and exercise (5), alongside medication to regulate plasma glucose levels and preserve kidney function (6). Early multifactorial intervention in patients with diabetes who also exhibit renal dysfunction is more likely to decrease the risk of associated severe complications (7, 8). Consequently, identifying effective, cost-efficient, and safe intervention strategies has become a critical necessity.

The study demonstrates that a deficiency in physical activity is associated with an elevated risk of various cardiovascular and metabolic diseases, notably diabetes mellitus (9). Glycemic fluctuations and insulin secretion in diabetic patients were significantly correlated with physical activity levels. Diabetic patients with low physical activity exhibited poorer glycemic control and reduced insulin secretion compared to those who consistently engaged in regular physical activity (10). Additionally, there was a negative correlation between physical activity and glycosylated hemoglobin (HbA1c) levels (11). In a Mendelian randomization study, it was found that a lack of physical activity increased the risk of diabetes; however, not all sedentary behaviors yielded this outcome (12). Physical activity serves not only to control the risk of diabetes but also offers protection against diabetic kidney disease. Fluctuations in urinary protein and renal function, key indicators of renal injury in diabetic kidney disease, have a strong association with physical activity. Moreover, a meta-analysis indicated that physical activity positively impacts estimated glomerular filtration rate (eGFR), decreases urinary albumin/creatinine ratio (UACR), and decelerates disease progression in diabetic kidney disease (13). Therefore, the KDIGO (Kidney Disease: Improving Global Outcomes) Diabetes Work Group recommends that patients with chronic kidney disease should engage in at least 150 minutes of moderate-intensity physical activity weekly (14).

Physical activity, a complex movement that generates energy expenditure, can typically be categorized into light, moderate, or vigorous based on the movement’s intensity. For patients with diabetic kidney disease, moderate-intensity physical activity, particularly aerobic exercise, is recommended as it modulates inflammatory cytokines, reduces oxidative stress, and improves renal function (14, 15). Physical activity can be categorized into different domains, namely occupation-related (OPA), transportation-related (TPA), and leisure-time physical activity (LTPA), based on its nature. Different domains of physical activity exert varying effects on diverse diseases. For instance, studies have shown that leisure-time physical activity (LTPA), exclusive of occupation-related (OPA) and transportation-related (TPA) activities, improves symptoms of depression (16). Additionally, all forms of physical activity have been found to reduce the risk of rectal cancer (17), while both TPA and LTPA are effective in alleviating muscular pain (18). Engagement in various domains of physical activity significantly reduces the risk of diabetes. Furthermore, a cross-sectional study indicated that, in addition to leisure-time physical activity (LTPA), other forms of physical activity also decrease the prevalence of diabetes across different racial categories (19). In another cohort study focusing on a Chinese population, high levels of occupation-related physical activity (OPA) were associated with a reduced risk of diabetes (20). While prior research has underscored the benefits of physical activity in managing diabetes, it remains uncertain whether different domains of physical activity (such as OPA, TPA, LTPA) exert identical effects on diabetic kidney disease.

In this study, our hypothesis posits that prolonged engagement in physical activity across all domains is associated with a reduced risk of diabetic kidney disease, and that this association would be strongly correlated with key kidney biomarkers (eGFR and UACR). This study aims to address the knowledge gap by exploring the relationship between various domains of physical activity, namely OPA, TPA, and LTPA, and diabetic kidney disease, utilizing data from the National Health and Nutrition Examination Survey (NHANES).

## 2 Methods

### 2.1 Study population

The study population was sourced from the National Health and Nutrition Examination Survey (NHANES), an extensive database that records the findings of a nationwide cross-sectional survey program implemented by the Centers for Disease Control and Prevention (CDC) (21). All participants provided informed consent at the time of the survey. The National Center for Health Statistics (NCHS) Research Ethics Review Board approved the survey protocol, negating the need for additional ethical review in this study. Cross-sectional data from six NHANES cycles, spanning 2007–2008, 2009–2010, 2011–2012, 2013–2014, 2015–2016, and 2017–2018, were selected for analysis.

**Figure 1:**
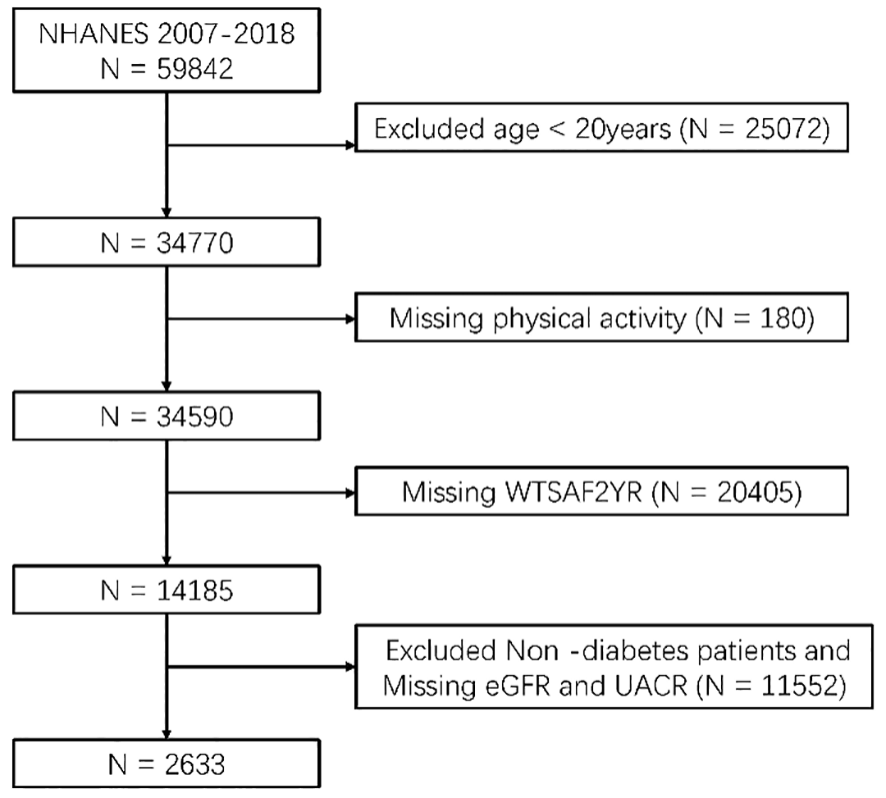
Flow diagram of the included survey participants.

Participants aged 20 years and older from the NHANES 2007–2018 cohort were included in the study. The exclusion criteria included: (1) People with missing physical activity data; (2) Non-diabetics; (3) People with missing kidney biomarkers (eGFR and UACR); (4) Missing weighting data (**Figure 1**).

### 2.2 Exposure variable

Physical activity data were collected from patients using the Global Physical Activity Questionnaire (GPAQ) (22). The questionnaire classified physical activity into domains such as OPA, TPA, and LTPA, capturing both the frequency (times per week) and duration (time per session) of these activities. OPA and LTPA need to differentiate between exercise intensities, namely vigorous and moderate exercise. Vigorous exercise typically involves activities that lead to significant increases in heart rate and entail high physical loads (e.g., lifting or carrying heavy objects, excavation, or construction work), whereas moderate exercise is characterized by activities with lighter loads that mildly affect heart rate variability (e.g., working with lighter objects). Duration of OPA and LTPA per week (h) = (number of vigorous physical activity sessions per week × duration of each vigorous physical activity session × 2 + number of moderate-intensity physical activity sessions per week × duration of each moderate-intensity physical activity session)/60 (19). The total physical activity, which was PA in this study, was the sum of OPA, TPA, and LTPA.

### 2.3 Outcome variable

A diabetes diagnosis is confirmed if any one of the following criteria is met: (1) Prior diabetes diagnosis; (2) Use of diabetes medication for plasma glucose control; (3) Fasting plasma glucose ≥7.0 mmol/L; (4) Glycosylated hemoglobin (HbA1c) ≥ 6.5%. Diabetic kidney disease is diagnosed when, in addition to diabetes, any of the following conditions are met (23): (1) eGFR (estimated glomerular filtration rate) < 60 mL/min/1.73m²; (2) ACR (Albumin-to-Creatinine Ratio) ≥ 30 mg/g. eGFR score calculations adhere to the guidelines of the National Kidney Foundation Laboratory Participation Workgroup for the CKD-EPI 2021 race-free equation (24).

### 2.4 Covariates

This study incorporated three categories of covariates: sociodemographic factors (age, sex, race/ethnicity, education level, poverty income ratio [PIR], marital status), lifestyle behaviors (smoking status, body mass index [BMI]), and chronic diseases (heart disease, hypertension, stroke, liver disease, cancer). Participants aged 20 years and older were included in this study, categorized into three age groups: young adults (20–44 years), middle-aged adults (45–64 years), and elderly (≥65 years). Racial categories included Mexican American, Other Hispanic, Non-Hispanic White, Non-Hispanic Black, and Other Race/Multi-Racial. Marital status categories were Married/living with partner, Never married, and Widowed/divorced. Educational levels were categorized as below high school, high school, and college or above. The poverty income ratio (PIR) was categorized into four levels: <1, 1–1.99, 2–3.99, ≥4. Smoking status was divided into three categories: Current smoker (daily and intermittent smokers), Former smoker (those who have smoked over 100 cigarettes in their lifetime but currently do not smoke), and Never smoker (those who have smoked fewer than 100 cigarettes in their lifetime). Body mass index (BMI) categories were defined as Low to normal (<25), Overweight (25–30), and Obese (≥30). The definitions of hypertension, stroke, liver disease, heart disease and cancer were based on a physician’s diagnosis or other health professional’s assessment. Based on the questionnaire data, conditions such as congestive heart failure, coronary heart disease, and angina pectoris were classified under the category of heart disease.

### 2.5 Statistical analyses

Data in NHANES are compiled from various items across multiple sections, inevitably resulting in some missing data. In this study, missing values were addressed using multiple imputation based on random forests (25). NHANES data collection involved complex sampling methods (21); therefore, this study utilized appropriately weighted samples for description and analysis. The differences between the two groups were compared by t-test or ANOVA in order to compare the characteristics of the study population between DKD(Patients with DKD) and non-DKD(Patients who do not have DKD). Continuous variables were presented as survey-weighted mean ± standard deviation, while categorical variables were expressed using frequencies and survey-weighted percentiles. The association between physical activity domains and the risk of diabetic kidney disease was assessed using survey-weighted logistic regression models. Similarly, the impact on kidney biomarkers was analyzed through survey-weighted generalized linear regression models, with all models adjusting for age and sex. Model 1 adjusted for sex and age only; Model 2, based on Model 1, additionally included race/ethnicity, education, PIR, marital status, smoking, and BMI; Model 3 included all aforementioned variables along with heart disease, hypertension, stroke, liver disease, and cancer. The nonlinear relationship between physical activity domains and eGFR, ACR was analyzed using survey-weighted restricted cubic spline (RCS), adjusting for all variables. Findings from the generalized linear regression were stratified according to sex, age, race/ethnicity, education, PIR, marital status, smoking, BMI, and chronic diseases such as heart disease, hypertension, stroke, liver disease, and cancer. Sensitivity analyses were conducted, adjusting for drinking status, hyperlipidemia, and respiratory disease, to assess the stability of the results with adjustments for all variables. All statistical analyses were conducted using R software (version 4.2.3; www.r-project.org), with a significance threshold set at P < 0.05.

## 3 Results

### 3.1 Characteristics of study population

The dataset, derived from NHANES 2007–2018, included 2,663 participants, with 52.8% male and 47.2% female. The average age was 59.17 years (± 13.64). Among the diabetic patients, 973 (33.1%) had diabetic kidney disease, while 1660 (66.9%) did not have diabetes. Significant differences were observed between the two groups in terms of age, marital status, education, PIR, hypertension, heart disease, stroke, and liver disease (all P < 0.05). There were no significant differences between the groups regarding sex, race/ethnicity, smoking, BMI, and cancer. Significant differences in physical activity, specifically in PA, OPA, and LTPA, were noted between the two groups (**Table 1**). **Supplementary Table S2** presents the unweighted data of participants with and without diabetic kidney disease (DKD).

**Table 1:** Characteristics of study population.

	DKD (N=973)	non-DKD (N=1660)	P-value
**Age, M (SD)**	63.44 (13.84)	57.06 (13.03)	<0.001
20–44	82 (11.2%)	247 (16.5%)	<0.001
45–64	336 (34.7%)	838 (53.4)	
≥65	555 (54.2%)	575 (30.1%)	
**Sex, n (%)**			0.873
female	449 (47.6%)	793 (47.1%)	
male	524 (52.4%)	867 (52.9%)	
**Race/ethnicity, n (%)**			0.477
Mexican American	177 (11.1%)	303 (9.8%)	
Other Hispanic	107 (6.1%)	215 (6.6%)	
Non-Hispanic White	359 (59.1%)	558 (61.3%)	
Non-Hispanic Black	236 (15.4%)	373 (13.6%)	
Other Race - Including Multi-Racial	94 (8.3%)	211 (8.6%)	
**Marital status, n (%)**			<0.001
Married/living with partner	577 (59.7%)	1068 (67.2%)	
Never married	73 (7.8%)	173 (11.1%)	
Widowed/divorced	343 (32.5%)	419 (21.7%)	
**Education, n (%)**			0.005
below high school	363 (26.8%)	530 (21.6%)	
college or above	382 (45.3%)	747 (54.7%)	
high school	228 (27.9%)	383 (23.7%)	
**PIR, n (%)**			0.001
<1	247 (19.0%)	362 (15.6%)	
1–1.99	322 (28.5%)	486 (23.1%)	
2–3.99	243 (30.7%)	456 (30.0%)	
≥4	161 (21.8%)	356 (31.3%)	
**Smoking, n (%)**			0.350
Current smoker	155 (14.7%)	269 (15.4%)	
Former smoker	466 (48.1%)	875 (51.5%)	
Never smoker	352 (37.2%)	516 (33.1%)	
**BMI, kg/m2, n (%)**			0.370
Low to normal (<25)	139 (13.0%)	223 (11.2%)	
Obese (25–30)	572 (62.9%)	940 (61.8%)	
Overweight (≥30)	262 (24.2%)	497 (27.0%)	
Chronic disease, n (%)			
**Hypertension**	723 (72.9%)	968 (59.2%)	<0.001
**Heart disease**	282 (28.1%)	538 (16.2%)	<0.001
**Cancer**	165 (17.8%)	205 (14.7%)	0.145
**Stroke**	111 (11.5%)	85 (4.8%)	<0.001
**Liver disease**	62 (4.6%)	135 (7.8%)	0.014
**OPA, M (SD)**	5.86 (15.21)	8.16 (19.72)	0.015
**TPA, M (SD)**	0.79 (3.56)	0.75 (2.77)	0.749
**LTPA, M (SD)**	1.39 (3.28)	1.98 (4.21)	0.003
**PA, M (SD)**	8.05 (16.99)	10.88 (20.88)	0.006

n is an unprofitable value; % and m (SD) are weighted value. DKD, diabetic kidney disease; PIR, poverty income ratio; BMI, body mass index; OPA, occupation-related physical activity; TPA, transportation-related physical activity; LTPA, leisure-time physical activity; PA, total physical activity.

### 3.2 The relationship between physical activity and DKD

**Figure 2:**
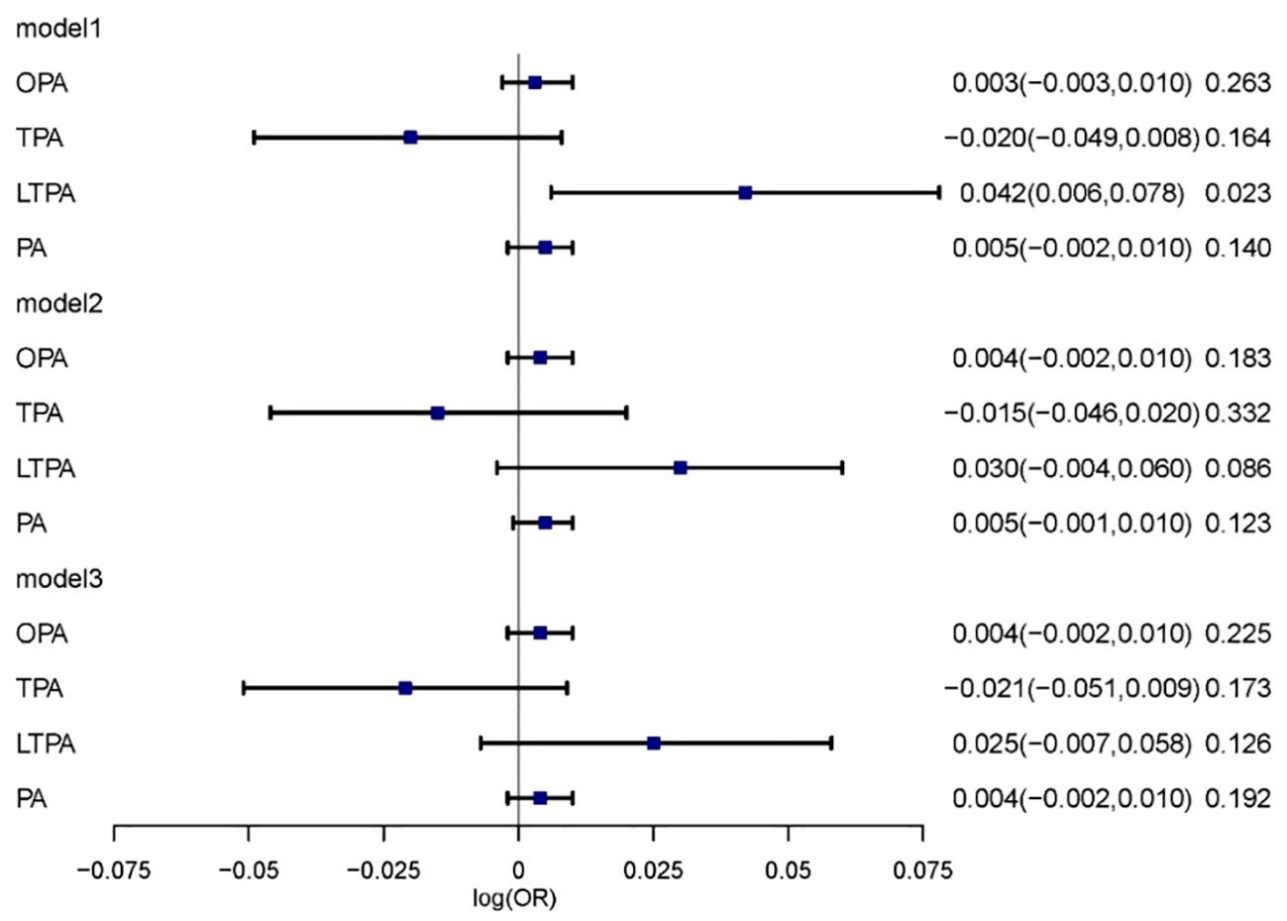
The relationship between physical activity and DKD. Multivariable model 1 was adjusted for age and sex; Multivariable model 2 was adjusted for age, sex, Race/ethnicity, Marital status, Education, PIR, Smoking and BMI. Multivariable model 3 was adjusted for age, sex, Race/ethnicity, Marital status, Education, PIR, Smoking, BMI, Hypertension, Heart, Cancer, Stroke and Liver. OPA, occupation-related physical activity; TPA, transportation-related physical activity; LTPA, leisure-time physical activity; PA, total physical activity.

Survey-weighted logistic regression models were employed to assess the correlation between various domains of physical activity and diabetic kidney disease. In Model 1, leisure-time physical activity (LTPA) was associated with an increased risk of diabetic kidney disease (DKD) (log[OR] = 0.042; 95% CI, 0.006–0.078, P = 0.023). However, when further adjusted for sociodemographic factors, lifestyle behaviors, and chronic diseases, the overall physical activity was not significantly associated with the development of DKD (**Figure 2**).

### 3.3 Linear relationship between physical activity and kidney biomarkers

**Figure 3:**
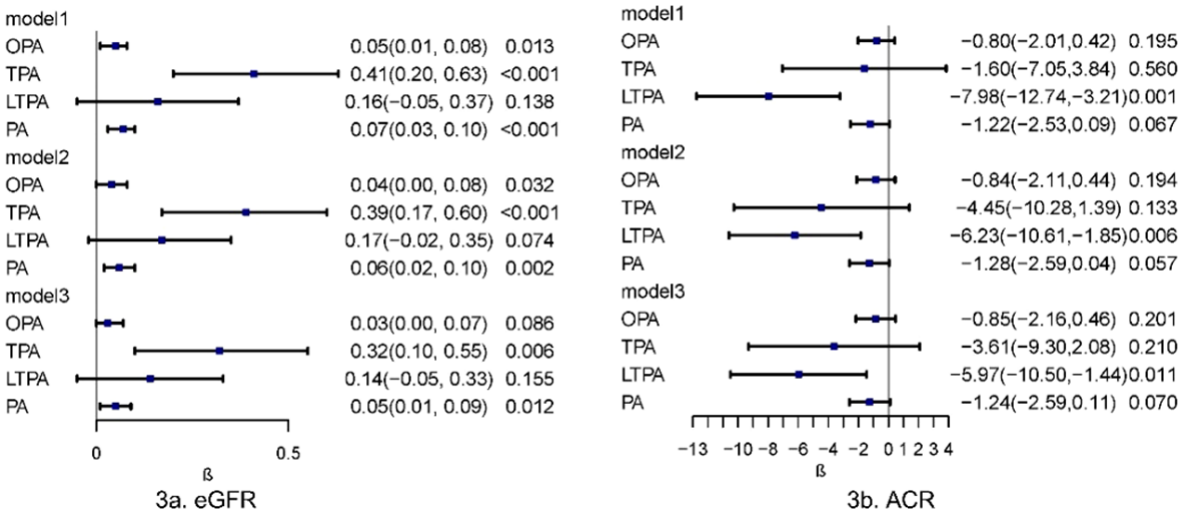
Multivariate linear regression analysis of physical activity in relation to kidney biomarkers. Multivariable model 1 was adjusted for age and sex; Multivariable model 2 was adjusted for age, sex, Race/ethnicity, Marital status, Education, PIR, Smoking and BMI. Multivariable model 3 was adjusted for age, sex, Race/ethnicity, Marital status, Education, PIR, Smoking, BMI, Hypertension, Heart, Cancer, Stroke and Liver. OPA, occupation-related physical activity; TPA, transportation-related physical activity; LTPA, leisure-time physical activity; PA, total physical activity; eGFR, estimated Glomerular Filtration Rate; ACR, Albumin/Urine Creatinine Ratio.

The associations between various physical activities and kidney biomarkers (eGFR and ACR) were analyzed using survey-weighted multiple linear regression. In Model 3, which was fully adjusted for sociodemographic factors, lifestyle behaviors, and chronic diseases, a positive correlation was observed between PA and eGFR (β = 0.05, 95% CI, 0.01–0.09, P = 0.012). A positive correlation was also found between TPA and eGFR (β = 0.32, 95% CI, 0.10–0.55, P = 0.006) in the fully adjusted models. A negative correlation was observed between LTPA and ACR (β = -5.97, 95% CI, -10.50 - -1.44, P = 0.011). A potential negative correlation between PA and ACR was suggested (β = -1.24, 95% CI, -2.59 - 0.11, P = 0.070), although this result was not definitively explained by linear regression (**Figure 3**).

### 3.4 Nonlinear relationship between physical activity and kidney biomarkers

**Figure 4:**
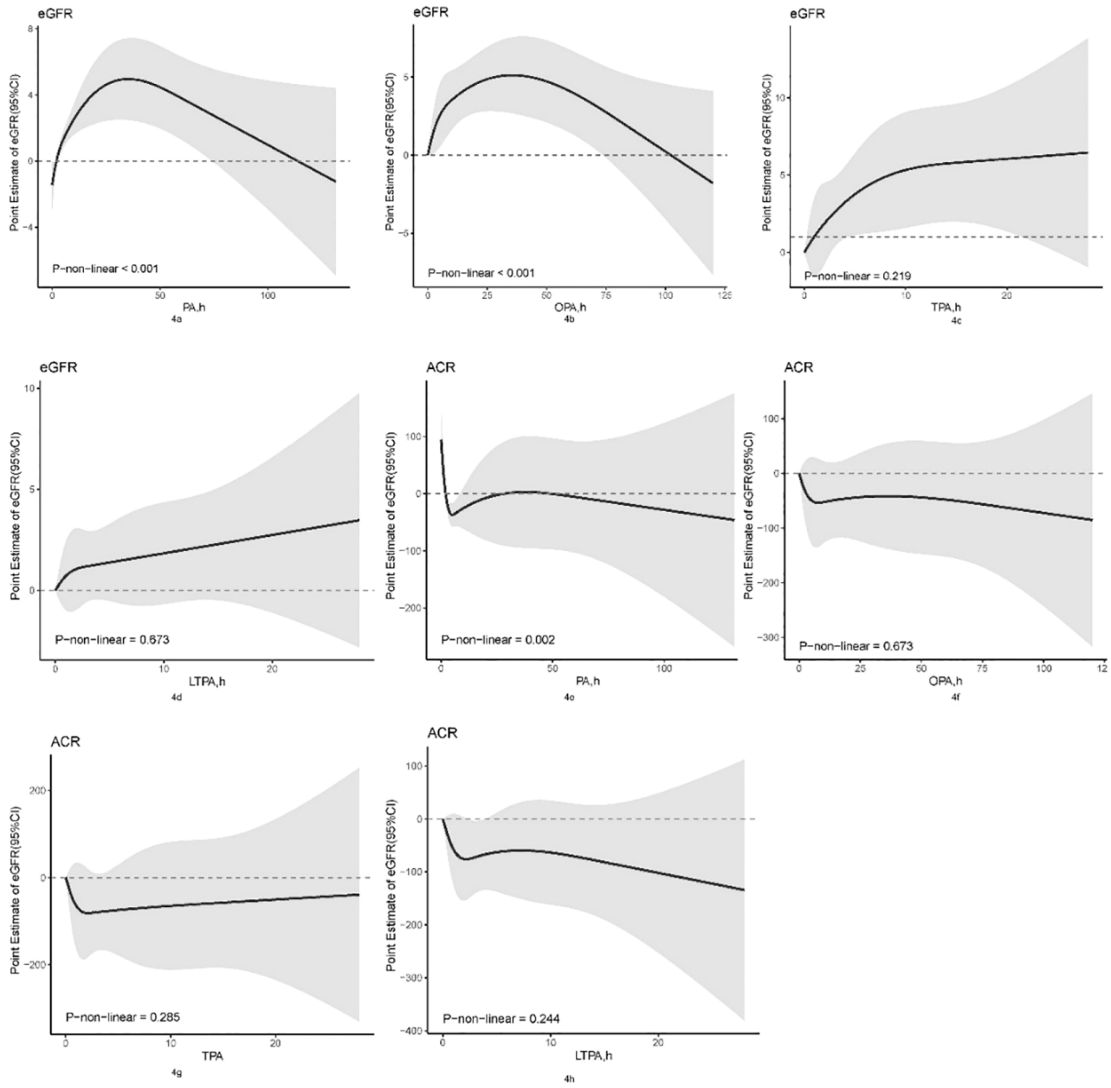
Non-linear association between physical activity and kidney biomarkers by the restricted cubic spline model. OPA, occupation-related physical activity; TPA, transportation-related physical activity; LTPA, leisure-time physical activity; PA, total physical activity; eGFR, estimated Glomerular Filtration Rate; ACR, Albumin/Urine Creatinine Ratio.

The nonlinear relationships between various physical activity domains and kidney biomarkers (eGFR and ACR) were depicted using restricted cubic spline analysis, as shown in **Figure 4**. In the RCS model, a nonlinear relationship was observed between PA and eGFR (**Figure 4**, P for non-linearity <0.001), where eGFR increased with physical activity duration up to 35.023 h/week, then plateaued. A nonlinear relationship was also found between OPA and eGFR (**Figure 4**, P for non-linearity <0.001), where eGFR peaked at 34.975 h/week of OPA and decreased subsequently. Nonlinear correlation between PA and ACR was noted (**Figure 4**, P for non-linearity = 0.002), where ACR first decreased to a low point at 5.287 h/week, slightly increased, and then continued to decrease after 37.006 h/week. There were no significant nonlinear correlations between other physical activities and kidney biomarkers.

### 3.5 Sensitivity analysis and subgroup analysis

**Supplementary Tables S4–8** display the stability of sensitivity analysis results in this study. Following adjustments for drinking status, hyperlipidemia, and respiratory disease, TPA and PA continued to show a positive correlation with eGFR, while LTPA maintained a negative correlation with ACR. Stratification by age, sex, race/ethnicity, education, PIR, marital status, smoking, BMI, and various chronic diseases was conducted to assess if subgroup differences influenced the correlation between physical activity and kidney biomarkers (**Supplementary Tables S9–11**). PA showed a significant association with eGFR in all strata except for the elderly (age ≥65 years); in the cancer stratum, this association was notably moderated. TPA exhibited significant associations with eGFR in subgroups including young adults (age 20–44 years), sex, race/ethnicity (Non-Hispanic White, Other Race/Multi-Racial), education, PIR, marital status (Married/living with partner, Widowed/divorced), smoking, BMI (Low to normal, Overweight), and the presence of heart disease, hypertension, but not stroke, liver disease, or cancer. A significant moderating effect was observed in the gender stratum.

## 4 Discussion

Most studies on physical activity and diabetes-related complications concentrate on microangiopathy, peripheral neuropathy, and cardiovascular events (26, 27), while fewer studies have examined, often with conflicting results, the relationship with diabetic kidney disease risk (13, 28). In this nationally representative cross-sectional study, no effect of physical activity on the progression from diabetes to diabetic kidney disease was observed after adjusting for sociodemographic factors, lifestyle behaviors, and chronic diseases. Previous research identified a link between leisure-time physical activity (LTPA) and diabetic kidney disease, emphasizing the role of vigorous physical activity levels. This study, which refined the classification of physical activity domains, did not find a correlation between the duration of OPA, TPA, and LTPA and the risk of developing diabetic kidney disease.

Although some studies have shown a correlation between physical activity and diabetic kidney disease (29, 30), this study further explored the relationship between physical activity and renal biomarkers (eGFR and ACR) across different domains. Following adjustments for age and sex, a positive correlation was observed between TPA and PA with eGFR, while LTPA showed a negative correlation with ACR. Upon further adjustments for race/ethnicity, education, PIR, marital status, smoking, BMI, and chronic diseases, the positive correlation of TPA and PA with eGFR persisted, as did the negative correlation of LTPA with ACR. This study elucidates the types of exercise within different domains of physical activity that are associated with a lower risk of kidney injury among patients with diabetic kidney disease. After comprehensive adjustments for all variables, it was found that managing appropriate durations of physical activity is beneficial in mitigating the risk of diabetic kidney disease. RCS results indicated that up to 34.975 hours per week of OPA positively correlates with eGFR, while overall PA was associated with increased eGFR and decreased ACR.

Appropriate patterns and durations of physical activity serve as protective factors against diabetic kidney disease. The mechanisms underlying how physical activity protects renal function in diabetic patients likely relate to its role in aiding better blood glucose control. Studies show that short-term reduction in physical activity in healthy individuals leads to significant increases in postprandial blood glucose and insulin sensitivity, whereas long-term inactivity can result in hepatic mitochondrial dysfunction and hepatic steatosis (31, 32). Short-term hyperglycemic states typically do not significantly impact healthy individuals, and transient elevations of blood glucose in non-diabetic patients during severe illness are generally considered harmless (33). In diabetic patients, persistent or frequent high glucose levels lead to high filtration states in the glomerulus, resulting in increased thylakoid matrix, glomerular thylakoid dilatation, and tubular fibrosis, progressively affecting the entire kidney (34). Diabetic individuals who remain physically active experience smoother blood glucose fluctuations, with physical activity duration inversely correlated with glucose variability (35). Physical activity aids individuals with diabetes in controlling blood glucose, lipids, and weight, and in reducing inflammation, cardiovascular events, and mortality (36). A certain level of physical activity is necessary for pregnant women during pregnancy. This study included pregnant women in the population, noting that moderate-intensity physical activity reduces the risk of gestational diabetes mellitus (37).

Limited research exists on the impact of various physical activity domains on diabetes complications, with most prior studies emphasizing the risk of diabetes associated with different physical activity areas (19, 20). Previous research on physical activity in relation to diabetes complications predominantly centered on the correlation between leisure-time physical activity (LTPA) or overall physical activity (PA) and various diseases. The FinnDiane Study Group, in a longitudinal observational study, observed that higher levels of LTPA, particularly frequent and intense activities, were associated with reduced cardiovascular event risks in type 1 diabetes patients (27). The team noted that vigorous LTPA was linked to a reduced risk of developing type 1 diabetic kidney disease, with minor relevance to the overall quantity and duration of LTPA as indicated by previous research (38). Aerobic LTPA, in any form, is widely recognized for reducing mortality risk in individuals with diabetes, while inactivity, particularly in non-Hispanic whites, significantly increases this risk (39). A prospective cohort study involving 445,075 participants revealed that individuals with diabetes adhering to or exceeding physical activity standards significantly reduced morbidity and mortality from chronic kidney disease. Additionally, physical activity in those with both conditions correlated with a reduction of 446.5 deaths per 100,000 people, compared to having diabetes or chronic kidney disease alone (40). Engaging in LTPA for over 30 minutes daily during pregnancy was found to lower the risk of gestational diabetes and pre-eclampsia in pregnant women, without adverse fetal effects (41). This study found no correlation between the overall duration of physical activity across all domains and the risk of developing diabetic kidney disease. However, significant correlations with eGFR and ACR were observed in diabetic kidney disease, and an optimal weekly physical activity duration was identified through RCS analysis. Consequently, the relationship between OPA, TPA, and LTPA and diabetic kidney disease remains controversial and warrants further exploration, particularly in terms of physical activity intensity and frequency.

The present study demonstrated a significant association between multiple domains of physical activity and markers of renal function in individuals with diabetes mellitus progressing to chronic kidney disease (CKD), particularly through moderate physical activity—defined as exercise that is lightly loaded and mildly affects heart rate variability. Our findings indicate that the optimal duration of exercise inflection point is 35 hours per week; thus, it is recommended that patients should not engage in more than 35 hours of moderate exercise weekly. According to the GPAQ, strenuous physical activity, which is highly loaded and significantly increases heart rate, is considered twice as demanding as moderate physical activity (22), and should therefore not exceed 17.5 hours per week. OPA accounts for a significant portion of daily activity, especially among patients with different occupational demands who spend more than one-third of their day working. We recommend adjusting work hours based on the intensity of occupational activities. Specifically, exercise intensity for occupational categories was classified as medium OPA for technicians, service workers, and machine operators, and high OPA for agricultural workers, craftsmen, and laborers (42). By integrating the results of this study with specific occupations, we guided diabetic patients in different professions to engage in appropriately timed OPA. For example, teachers, police officers, and truck drivers should not exceed 35 hours per week, whereas construction workers, logisticians, and forestry workers should not exceed 17.5 hours per week. Previous research suggests that exercise can lead to dehydration and an elevation of core body temperature, which may trigger insufficient renal perfusion, potentially harming the kidneys and causing elevated serum creatinine levels (43). However, it is important to note that appropriate exercise does not harm the kidneys; only prolonged exercise contributes to a decrease in eGFR (44), echoing our study’s findings.

Our study examined the correlation between the duration of physical activity across different domains and the risk of diabetic kidney disease in the U.S. population, as per NHANES data. Initially, we controlled for the effects of various confounders on outcomes by adjusting for sociodemographic factors, lifestyle behaviors, and chronic diseases. Secondly, we analyzed the correlation between various physical activity domains and diabetic kidney disease using multiple statistical methods, assessing the results’ stability through sensitivity analyses. Remaining gaps in our study include: 1. While distinguishing between different physical activity domains, the focus was on activity duration. Future analyses should consider the frequency and intensity of physical activity. 2. The cross-sectional nature of our data limits causal inference; future longitudinal studies should explore causality in physical activity domains and potential confounders. 3. Despite controlling for numerous confounders and adjusting for factors like drinking status, hyperlipidemia, and respiratory disease, other confounders may have influenced the conclusions.

## 5 Conclusions

Our study did not establish a direct association between the duration of physical activity in each domain and the risk of diabetic kidney disease. Nevertheless, various physical activities showed linear correlations with renal biomarkers (e.g., TPA with eGFR, LTPA, PA with ACR), and certain domains of activity exhibited nonlinear correlations with eGFR and ACR. It was suggested that an exercise duration not exceeding 35 hours per week is associated with a lower risk of renal impairment in patients with diabetic kidney disease. Synthesizing the findings of our analyses, we propose that total physical activity (TPA) and occupational physical activity (OPA) are preferable for diabetic patients with reduced eGFR, while leisure-time physical activity (LTPA) is particularly recommended for those with elevated ACR levels. This cross-sectional study initially identifies an association between diverse exercise domains and decreased risk of renal impairment. However, future longitudinal studies employing objective measurements of physical activity are warranted to corroborate and extend our findings. Meanwhile, follow-up studies should expand the age range of the population to include pediatric subjects to subdivide the diabetes subtypes and provide clinical guidance for different domains of physical activity to control renal impairment in patients with different subtypes of diabetes.

## Data availability statement

Publicly available datasets were analyzed in this study. This data can be found here: https://www.cdc.gov/nchs/nhanes/index.htm.

## Ethics statement

The studies involving humans were approved by National Center for Health Statistics. The studies were conducted in accordance with the local legislation and institutional requirements. The participants provided their written informed consent to participate in this study.

## Author contributions

PH: Writing – original draft, Writing – review & editing. YD: Conceptualization, Writing – review & editing. SD: Data curation, Writing – review & editing. HL: Data curation, Writing – review & editing. CL: Formal analysis, Writing – review & editing. YM: Methodology, Writing – review & editing. CT: Methodology, Writing – review & editing. MZ: Funding acquisition, Supervision, Writing – review & editing.
