# Supplementary material for: Association of different domains of physical activity with diabetic kidney disease: a population-based study
Source: Front Endocrinol (Lausanne). 2024 May 28;15:1364028. doi: 10.3389/fendo.2024.1364028 (PMC11165133; doi:10.3389/fendo.2024.1364028)
Supplement: Supplementary file 1 [file DataSheet_1.docx]

Supplementary Material

# Supplementary Table S1. Missing data patterns.

| Pattern | N | % |
| --- | --- | --- |
| nothing | 390 | 14.81 |
| DMDEDUC2 | 3 | 0.11 |
| INDFMPIR | 261 | 9.91 |
| DMDMARTL | 2 | 0.08 |
| SMO | 2 | 0.08 |
| BMXBMI | 38 | 1.44 |
| MCQ160B | 11 | 0.42 |
| MCQ160C | 24 | 0.91 |
| MCQ160D | 18 | 0.68 |
| MCQ160E | 8 | 0.30 |
| MCQ160F | 4 | 0.15 |
| MCQ160L | 11 | 0.42 |
| MCQ220 | 6 | 0.23 |
| BPQ020 | 2 | 0.08 |

# Supplementary Figure S1. Data situation diagram before and after multiple imputation.


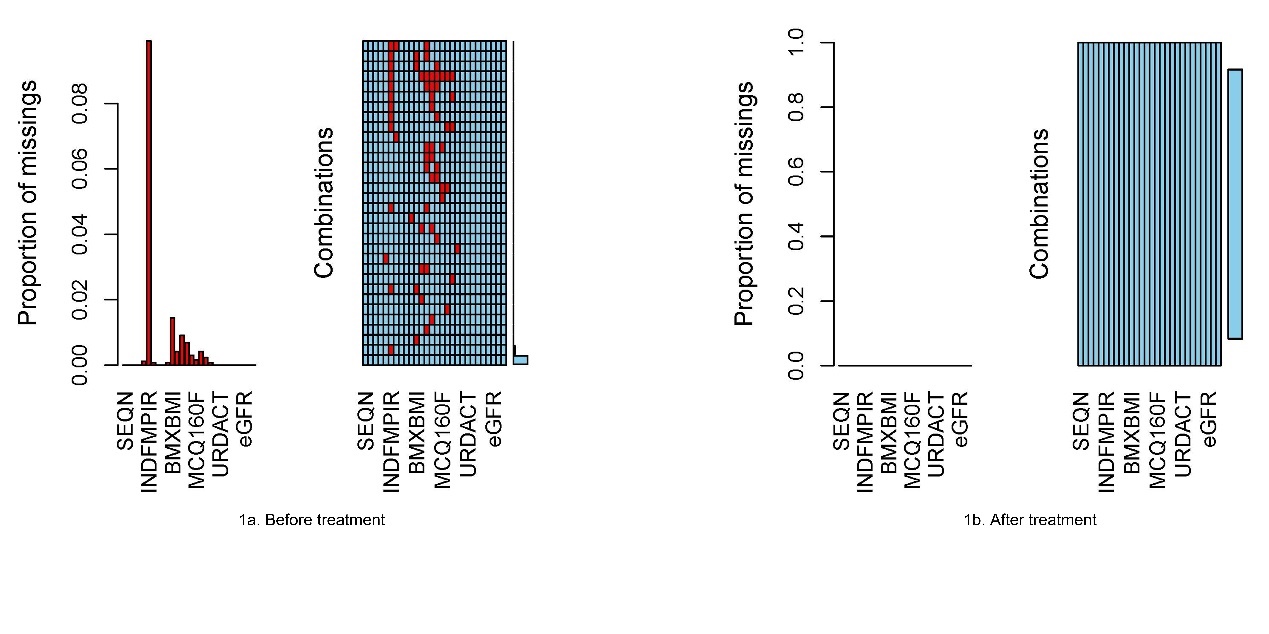


# Supplementary Table S2. The research on unweighted population characteristics.

|  | **DKD (N=973)** | **non-DKD (N=1660)** | **Overall (N=2633)** | **P-value** |
| --- | --- | --- | --- | --- |
| **Sex, n (%)** |  |  |  |  |
| female | 449 (46.1%) | 793 (47.8%) | 1242 (47.2%) | 0.7 |
| male | 524 (53.9%) | 867 (52.2%) | 1391 (52.8%) |  |
| **Age，M (SD)** |  |  |  |  |
| 20–44 | 555 (57.0%) | 575 (34.6%) | 1130 (42.9%) | <0.001 |
| 45–64 | 82 (8.4%) | 247 (14.9%) | 329 (12.5%) |  |
| ≥65 | 336 (34.5%) | 838 (50.5%) | 1174 (44.6%) |  |
| **Race/ethnicity, n (%)** |  |  |  |  |
| Mexican American | 177 (18.2%) | 303 (18.3%) | 480 (18.2%) | 0.3 |
| Other Hispanic | 107 (11.0%) | 215 (13.0%) | 322 (12.2%) |  |
| Non-Hispanic White | 359 (36.9%) | 558 (33.6%) | 917 (34.8%) |  |
| Non-Hispanic Black | 236 (24.3%) | 373 (22.5%) | 609 (23.1%) |  |
| Other Race - Including Multi-Racial | 94 (9.7%) | 211 (12.7%) | 305 (11.6%) |  |
| **Marital status, n (%)** |  |  |  |  |
| Married/living with partner | 557 (57.2%) | 1068 (64.3%) | 1625 (61.7%) | <0.001 |
| Never married | 73 (7.5%) | 173 (10.4%) | 246 (9.3%) |  |
| Widowed/divorced | 343 (35.3%) | 419 (25.2%) | 762 (28.9%) |  |
| **Education, n (%)** |  |  |  |  |
| below high school | 363 (37.3%) | 530 (31.9%) | 893 (33.9%) | 0.04 |
| college or above | 382 (39.3%) | 747 (45.0%) | 1129 (42.9%) |  |
| high school | 228 (23.4%) | 383 (23.1%) | 611 (23.2%) |  |
| **PIR, n (%)** |  |  |  |  |
| <1 | 247 (25.4%) | 362 (21.8%) | 609 (23.1%) | 0.02 |
| 1–1.99 | 322 (33.1%) | 486 (29.3%) | 808 (30.7%) |  |
| 2–3.99 | 243 (25.0%) | 456 (27.5%) | 699 (26.5%) |  |
| ≥4 | 161 (16.5%) | 356 (21.4%) | 517 (19.6%) |  |
| **Smoking, n (%)** |  |  |  |  |
| Current smoker | 155 (15.9%) | 269 (16.2%) | 424 (16.1%) | 0.1 |
| Former smoker | 466 (47.9%) | 875 (52.7%) | 1341 (50.9%) |  |
| Never smoker | 352 (36.2%) | 516 (31.1%) | 868 (33.0%) |  |
| **BMI, kg/m2, n (%)** |  |  |  |  |
| Low to normal (<25) | 139 (14.3%) | 223 (13.4%) | 362 (13.7%) | 0.6 |
| Obese (25–30) | 572 (58.8%) | 940 (56.6%) | 1512 (57.4%) |  |
| Overweight (≥30) | 262 (26.9%) | 497 (29.9%) | 759 (28.8%) |  |
| **Hypertension, n (%)** |  |  |  |  |
| no | 250 (25.7%) | 692 (41.7%) | 942 (35.8%) | <0.001 |
| yes | 723 (74.3%) | 968 (58.3%) | 1691 (64.2%) |  |
| **Heart, n (%)** |  |  |  |  |
| no | 691 (71.0%) | 1404 (84.6%) | 2095 (79.6%) | <0.001 |
| yes | 282 (29.0%) | 256 (15.4%) | 538 (20.4%) |  |
| **Stroke, n (%)** |  |  |  |  |
| no | 862 (88.6%) | 1575 (94.9%) | 2437 (92.6%) | <0.001 |
| yes | 111 (11.4%) | 85 (5.1%) | 196 (7.4%) |  |
| **Liver, n (%)** |  |  |  |  |
| no | 911 (93.6%) | 1525 (91.9%) | 2436 (92.5%) | 0.3 |
| yes | 62 (6.4%) | 135 (8.1%) | 197 (7.5%) |  |
| **Cancer, n (%)** |  |  |  |  |
| no | 808 (83.0%) | 1455 (87.7%) | 2263 (85.9%) | 0.005 |
| yes | 165 (17.0%) | 205 (12.3%) | 370 (14.1%) |  |
| **OPA，Mean (SD)** | 5.71 (15.4) | 7.50 (19.9) | 6.84 (18.3) | 0.08 |
| **TPA，Mean (SD)** | 1.01 (3.81) | 0.983 (3.44) | 0.994 (3.58) | 0.2 |
| **LTPA，Mean (SD)** | 1.33 (3.42) | 1.87 (4.25) | 1.67 (3.97) | <0.001 |
| **PA，Mean (SD)** | 8.05 (17.1) | 10.4 (21.2) | 9.50 (19.8) | <0.001 |

*Note: DKD, diabetic kidney disease; PIR , poverty income ratio; BMI, body mass index; OPA, occupation-related physical activity; TPA, transportation-related physical activity; LTPA, leisure-time physical activity; PA, total physical activity.*

# Supplementary Table S3. Sensitivity analysis of the association between PA and the risk of DKD.

| **Characteristic** | **log(OR)** | **95% CI** | **p-value** |
| --- | --- | --- | --- |
| **Sex, n (%)** |  |  |  |
| female | — | — |  |
| male | -0.15 | -0.43, 0.14 | 0.3 |
| **Age，M (SD)** |  |  |  |
| 20–44 | — | — |  |
| 45–64 | 0.78 | 0.37, 1.2 | <0.001 |
| ≥65 | 1 | 0.72, 1.3 | <0.001 |
| **Race/ethnicity, n (%)** |  |  |  |
| Mexican American | — | — |  |
| Other Hispanic | 0.26 | -0.11, 0.63 | 0.2 |
| Non-Hispanic White | 0.42 | 0.11, 0.73 | 0.009 |
| Non-Hispanic Black | 0.16 | -0.14, 0.47 | 0.3 |
| Other Race - Including Multi-Racial | 0.27 | -0.13, 0.67 | 0.2 |
| **Marital status, n (%)** |  |  |  |
| Married/living with partner | — | — |  |
| Never married | -0.04 | -0.51, 0.43 | 0.9 |
| Widowed/divorced | -0.31 | -0.61, -0.02 | 0.038 |
| **Education, n (%)** |  |  |  |
| below high school | — | — |  |
| college or above | 0.05 | -0.32, 0.42 | 0.8 |
| high school | -0.14 | -0.43, 0.15 | 0.3 |
| **PIR, n (%)** |  |  |  |
| <1 | — | — |  |
| 1–1.99 | 0.07 | -0.23, 0.37 | 0.6 |
| 2–3.99 | 0.07 | -0.30, 0.44 | 0.7 |
| ≥4 | 0.28 | -0.10, 0.65 | 0.14 |
| **Smoking, n (%)** |  |  |  |
| Current smoker | — | — |  |
| Former smoker | 0.21 | -0.17, 0.59 | 0.3 |
| Never smoker | 0.13 | -0.23, 0.50 | 0.5 |
| **BMI, kg/m2, n (%)** |  |  |  |
| Low to normal (<25) | — | — |  |
| Obese (25–30) | 0.04 | -0.31, 0.39 | 0.8 |
| Overweight (≥30) | 0.24 | -0.14, 0.61 | 0.2 |
| **Hypertension, n (%)** |  |  |  |
| no | — | — |  |
| yes | -0.55 | -0.84, -0.26 | <0.001 |
| **Heart, n (%)** |  |  |  |
| no | — | — |  |
| yes | -0.34 | -0.67, -0.01 | 0.044 |
| **Stroke, n (%)** |  |  |  |
| no | — | — |  |
| yes | -0.53 | -1.0, -0.06 | 0.029 |
| **Liver, n (%)** |  |  |  |
| no | — | — |  |
| yes | 0.67 | 0.17, 1.2 | 0.009 |
| **Cancer, n (%)** |  |  |  |
| no | — | — |  |
| yes | 0.13 | -0.21, 0.47 | 0.4 |
| **Hyperlipidemia, n (%)** |  |  |  |
| no | — | — |  |
| yes | -0.4 | -0.64, -0.16 | 0.001 |
| **Respiratory, n (%)** |  |  |  |
| no | — | — |  |
| yes | -0.02 | -0.27, 0.23 | 0.9 |
| **Drinke, n (%)** |  |  |  |
| Heavy drinker | — | — |  |
| Low to moderate drinker | 0.19 | -0.09, 0.48 | 0.2 |
| Nondrinker | -0.3 | -0.58, -0.03 | 0.031 |
| **PA，Mean (SD)** | 0 | 0.00, 0.01 | 0.2 |

*Note: DKD, diabetic kidney disease; PIR , poverty income ratio; BMI, body mass index; PA, total physical activity.*

# Supplementary Table S4. Sensitivity analysis of the association between OPA/TPA/LTPA and the risk of DKD.

| **Characteristic** | **log(OR)** | **95% CI** | **p-value** |
| --- | --- | --- | --- |
| **Sex, n (%)** |  |  |  |
| female | — | — |  |
| male | -0.15 | -0.43, 0.13 | 0.3 |
| **Age，M (SD)** |  |  |  |
| 20–44 | — | — |  |
| 45–64 | 0.76 | 0.35, 1.2 | <0.001 |
| ≥65 | 1 | 0.72, 1.3 | <0.001 |
| **Race/ethnicity, n (%)** |  |  |  |
| Mexican American | — | — |  |
| Other Hispanic | 0.28 | -0.09, 0.65 | 0.13 |
| Non-Hispanic White | 0.42 | 0.11, 0.74 | 0.008 |
| Non-Hispanic Black | 0.17 | -0.13, 0.47 | 0.3 |
| Other Race - Including Multi-Racial | 0.27 | -0.14, 0.67 | 0.2 |
| **Marital status, n (%)** |  |  |  |
| Married/living with partner | — | — |  |
| Never married | -0.03 | -0.50, 0.44 | 0.9 |
| Widowed/divorced | -0.31 | -0.61, -0.02 | 0.038 |
| **Education, n (%)** | |  |  |
| below high school | — | — |  |
| college or above | 0.03 | -0.34, 0.39 | 0.9 |
| high school | -0.16 | -0.44, 0.13 | 0.3 |
| **PIR, n (%)** |  |  |  |
| <1 | — | — |  |
| 1–1.99 | 0.06 | -0.24, 0.37 | 0.7 |
| 2–3.99 | 0.06 | -0.31, 0.43 | 0.7 |
| ≥4 | 0.26 | -0.12, 0.64 | 0.2 |
| **Smoking, n (%)** |  |  |  |
| Current smoker | — | — |  |
| Former smoker | 0.2 | -0.17, 0.58 | 0.3 |
| Never smoker | 0.11 | -0.24, 0.47 | 0.5 |
| **BMI, kg/m2, n (%)** |  |  |  |
| Low to normal (<25) | — | — |  |
| Obese (25–30) | 0.04 | -0.32, 0.39 | 0.8 |
| Overweight (≥30) | 0.22 | -0.16, 0.60 | 0.2 |
| **Hypertension, n (%)** | |  |  |
| no | — | — |  |
| yes | -0.55 | -0.85, -0.26 | <0.001 |
| **Heart, n (%)** |  |  |  |
| no | — | — |  |
| yes | -0.34 | -0.67, -0.01 | 0.041 |
| **Stroke, n (%)** |  |  |  |
| no | — | — |  |
| yes | -0.53 | -1.0, -0.05 | 0.029 |
| **Liver, n (%)** |  |  |  |
| no | — | — |  |
| yes | 0.66 | 0.16, 1.2 | 0.01 |
| **Cancer, n (%)** |  |  |  |
| no | — | — |  |
| yes | 0.12 | -0.22, 0.46 | 0.5 |
| **Hyperlipidemia, n (%)** | |  |  |
| no | — | — |  |
| yes | -0.4 | -0.64, -0.16 | 0.001 |
| **Respiratory, n (%)** | |  |  |
| no | — | — |  |
| yes | -0.03 | -0.28, 0.22 | 0.8 |
| **Drinke, n (%)** |  |  |  |
| Heavy drinker | — | — |  |
| Low to moderate drinker | 0.18 | -0.11, 0.46 | 0.2 |
| Nondrinker | -0.32 | -0.60, -0.04 | 0.025 |
| **OPA，Mean (SD)** | 0 | 0.00, 0.01 | 0.3 |
| **TPA，Mean (SD)** | -0.02 | -0.05, 0.01 | 0.2 |
| **LTPA，Mean (SD)** | 0.02 | -0.01, 0.06 | 0.13 |

*Note: DKD, diabetic kidney disease; PIR , poverty income ratio; BMI, body mass index; OPA, occupation-related physical activity; TPA, transportation-related physical activity; LTPA, leisure-time physical activity.*

# Supplementary Table S5. Sensitivity analysis of the association between PA and eGFR.

| **Characteristic** | **β** | **95% CI** | **p-value** |
| --- | --- | --- | --- |
| **Sex, n (%)** |  |  |  |
| female | — | — |  |
| male | -1.7 | -3.8, 0.36 | 0.1 |
| **Age，M (SD)** |  |  |  |
| 20–44 | — | — |  |
| 45–64 | 33 | 30, 36 | <0.001 |
| ≥65 | 17 | 15, 20 | <0.001 |
| **Race/ethnicity, n (%)** |  |  |  |
| Mexican American | — | — |  |
| Other Hispanic | -2.9 | -5.5, -0.40 | 0.024 |
| Non-Hispanic White | -6.3 | -8.9, -3.7 | <0.001 |
| Non-Hispanic Black | -13 | -15, -10 | <0.001 |
| Other Race - Including Multi-Racial | -2 | -5.1, 1.1 | 0.2 |
| **Marital status, n (%)** |  |  |  |
| Married/living with partner | — | — |  |
| Never married | -1 | -5.1, 3.0 | 0.6 |
| Widowed/divorced | -4.8 | -7.3, -2.4 | <0.001 |
| **Education, n (%)** | |  |  |
| below high school | — | — |  |
| college or above | -2.9 | -5.2, -0.69 | 0.011 |
| high school | -3.6 | -6.4, -0.81 | 0.012 |
| **PIR, n (%)** |  |  |  |
| <1 | — | — |  |
| 1–1.99 | 1.1 | -1.9, 4.0 | 0.5 |
| 2–3.99 | 1.2 | -1.6, 4.1 | 0.4 |
| ≥4 | 1.2 | -1.9, 4.3 | 0.5 |
| **Smoking, n (%)** |  |  |  |
| Current smoker | — | — |  |
| Former smoker | -3.8 | -6.4, -1.2 | 0.005 |
| Never smoker | -2.2 | -4.9, 0.58 | 0.12 |
| **BMI, kg/m2, n (%)** |  |  |  |
| Low to normal (<25) | — | — |  |
| Obese (25–30) | 1.2 | -2.4, 4.9 | 0.5 |
| Overweight (≥30) | 1.2 | -2.4, 4.8 | 0.5 |
| **Hypertension, n (%)** | |  |  |
| no | — | — |  |
| yes | -5.1 | -7.3, -3.0 | <0.001 |
| **Heart, n (%)** |  |  |  |
| no | — | — |  |
| yes | -4.6 | -7.3, -2.0 | <0.001 |
| **Stroke, n (%)** |  |  |  |
| no | — | — |  |
| yes | -5.3 | -10, -0.61 | 0.027 |
| **Liver, n (%)** |  |  |  |
| no | — | — |  |
| yes | 3 | -0.61, 6.7 | 0.1 |
| **Cancer, n (%)** |  |  |  |
| no | — | — |  |
| yes | -2.7 | -5.3, -0.06 | 0.045 |
| **Hyperlipidemia, n (%)** | |  |  |
| no | — | — |  |
| yes | -0.69 | -2.5, 1.1 | 0.5 |
| **Respiratory, n (%)** | |  |  |
| no | — | — |  |
| yes | 0.87 | -1.2, 3.0 | 0.4 |
| **Drinke, n (%)** |  |  |  |
| Heavy drinker | — | — |  |
| Low to moderate drinker | -2.1 | -4.1, -0.04 | 0.045 |
| Nondrinker | -8.1 | -11, -5.0 | <0.001 |
| **PA，Mean (SD)** | 0.05 | 0.01, 0.09 | 0.022 |

*Note: eGFR, estimated Glomerular Filtration Rate; PIR , poverty income ratio; BMI, body mass index; PA, total physical activity.*

# Supplementary Table S6. Sensitivity analysis of the association between OPA/TPA/LTPA and eGFR.

| **Characteristic** | **β** | **95% CI** | **p-value** |
| --- | --- | --- | --- |
| **Sex, n (%)** |  |  |  |
| female | — | — |  |
| male | -1.8 | -3.9, 0.27 | 0.087 |
| **Age，M (SD)** |  |  |  |
| 20–44 | — | — |  |
| 45–64 | 33 | 30, 36 | <0.001 |
| ≥65 | 17 | 15, 20 | <0.001 |
| **Race/ethnicity, n (%)** |  |  |  |
| Mexican American | — | — |  |
| Other Hispanic | -2.9 | -5.5, -0.39 | 0.025 |
| Non-Hispanic White | -6 | -8.6, -3.4 | <0.001 |
| Non-Hispanic Black | -13 | -15, -9.9 | <0.001 |
| Other Race - Including Multi-Racial | -1.9 | -4.9, 1.2 | 0.2 |
| **Marital status, n (%)** |  |  |  |
| Married/living with partner | — | — |  |
| Never married | -1.1 | -5.1, 2.9 | 0.6 |
| Widowed/divorced | -4.8 | -7.3, -2.4 | <0.001 |
| **Education, n (%)** | |  |  |
| below high school | — | — |  |
| college or above | -3.1 | -5.4, -0.80 | 0.009 |
| high school | -3.7 | -6.4, -0.88 | 0.011 |
| **PIR, n (%)** |  |  |  |
| <1 | — | — |  |
| 1–1.99 | 1.2 | -1.7, 4.1 | 0.4 |
| 2–3.99 | 1.3 | -1.5, 4.2 | 0.3 |
| ≥4 | 1.2 | -1.9, 4.3 | 0.4 |
| **Smoking, n (%)** |  |  |  |
| Current smoker | — | — |  |
| Former smoker | -3.9 | -6.5, -1.3 | 0.004 |
| Never smoker | -2.3 | -5.0, 0.50 | 0.11 |
| **BMI, kg/m2, n (%)** |  |  |  |
| Low to normal (<25) | — | — |  |
| Obese (25–30) | 1.4 | -2.3, 5.0 | 0.5 |
| Overweight (≥30) | 1.3 | -2.3, 4.8 | 0.5 |
| **Hypertension, n (%)** | |  |  |
| no | — | — |  |
| yes | -5 | -7.2, -2.9 | <0.001 |
| **Heart, n (%)** |  |  |  |
| no | — | — |  |
| yes | -4.7 | -7.3, -2.0 | <0.001 |
| **Stroke, n (%)** |  |  |  |
| no | — | — |  |
| yes | -5.1 | -9.8, -0.47 | 0.031 |
| **Liver, n (%)** |  |  |  |
| no | — | — |  |
| yes | 3 | -0.60, 6.7 | 0.1 |
| **Cancer, n (%)** |  |  |  |
| no | — | — |  |
| yes | -2.7 | -5.3, -0.05 | 0.046 |
| **Hyperlipidemia, n (%)** | |  |  |
| no | — | — |  |
| yes | -0.76 | -2.6, 1.1 | 0.4 |
| **Respiratory, n (%)** | |  |  |
| no | — | — |  |
| yes | 0.92 | -1.2, 3.0 | 0.4 |
| **Drinke, n (%)** |  |  |  |
| Heavy drinker | — | — |  |
| Low to moderate drinker | -2.2 | -4.2, -0.16 | 0.035 |
| Nondrinker | -8.1 | -11, -5.0 | <0.001 |
| **OPA，Mean (SD)** | 0.03 | -0.01, 0.07 | 0.14 |
| **TPA，Mean (SD)** | 0.3 | 0.07, 0.52 | 0.011 |
| **LTPA，Mean (SD)** | 0.15 | -0.05, 0.36 | 0.13 |

*Note: eGFR, estimated Glomerular Filtration Rate; PIR , poverty income ratio; BMI, body mass index; OPA, occupation-related physical activity; TPA, transportation-related physical activity; LTPA, leisure-time physical activity.*

# Supplementary Table S7. Sensitivity analysis of the association between PA and ACR.

| **Characteristic** | **β** | **95% CI** | **p-value** |
| --- | --- | --- | --- |
| **Sex, n (%)** |  |  |  |
| female | — | — |  |
| male | 52 | -27, 130 | 0.2 |
| **Age，M (SD)** |  |  |  |
| 20–44 | — | — |  |
| 45–64 | 39 | -52, 130 | 0.4 |
| ≥65 | 17 | -51, 85 | 0.6 |
| **Race/ethnicity, n (%)** |  |  |  |
| Mexican American | — | — |  |
| Other Hispanic | 62 | -86, 210 | 0.4 |
| Non-Hispanic White | -22 | -104, 61 | 0.6 |
| Non-Hispanic Black | 86 | -52, 223 | 0.2 |
| Other Race - Including Multi-Racial | 74 | -82, 230 | 0.3 |
| **Marital status, n (%)** |  |  |  |
| Married/living with partner | — | — |  |
| Never married | 27 | -82, 137 | 0.6 |
| Widowed/divorced | 15 | -73, 102 | 0.7 |
| **Education, n (%)** | |  |  |
| below high school | — | — |  |
| college or above | 32 | -53, 117 | 0.5 |
| high school | 46 | -61, 152 | 0.4 |
| **PIR, n (%)** |  |  |  |
| <1 | — | — |  |
| 1–1.99 | -152 | -272, -31 | 0.015 |
| 2–3.99 | -181 | -318, -44 | 0.01 |
| ≥4 | -220 | -350, -90 | 0.001 |
| **Smoking, n (%)** |  |  |  |
| Current smoker | — | — |  |
| Former smoker | -26 | -155, 102 | 0.7 |
| Never smoker | -44 | -167, 80 | 0.5 |
| **BMI, kg/m2, n (%)** |  |  |  |
| Low to normal (<25) | — | — |  |
| Obese (25–30) | -28 | -129, 74 | 0.6 |
| Overweight (≥30) | -29 | -123, 65 | 0.5 |
| **Hypertension, n (%)** | |  |  |
| no | — | — |  |
| yes | 112 | 51, 174 | <0.001 |
| **Heart, n (%)** |  |  |  |
| no | — | — |  |
| yes | 42 | -57, 141 | 0.4 |
| **Stroke, n (%)** |  |  |  |
| no | — | — |  |
| yes | -43 | -136, 49 | 0.4 |
| **Liver, n (%)** |  |  |  |
| no | — | — |  |
| yes | -8.5 | -151, 134 | >0.9 |
| **Cancer, n (%)** |  |  |  |
| no | — | — |  |
| yes | 19 | -77, 114 | 0.7 |
| **Hyperlipidemia, n (%)** | |  |  |
| no | — | — |  |
| yes | 102 | 22, 182 | 0.013 |
| **Respiratory, n (%)** | |  |  |
| no | — | — |  |
| yes | -0.32 | -70, 69 | >0.9 |
| **Drinke, n (%)** |  |  |  |
| Heavy drinker | — | — |  |
| Low to moderate drinker | -24 | -96, 49 | 0.5 |
| Nondrinker | 67 | -52, 186 | 0.3 |
| **PA，Mean (SD)** | -1.2 | -2.5, 0.11 | 0.071 |

*Note: ACR, Albumin/Urine Creatinine Ratio; PIR , poverty income ratio; BMI, body mass index; PA, total physical activity.*

# Supplementary Table S8. Sensitivity analysis of the association between OPA/TPA/LTPA and ACR.

| **Characteristic** | **β** | **95% CI** | **p-value** |
| --- | --- | --- | --- |
| **Sex, n (%)** |  |  |  |
| female | — | — |  |
| male | 54 | -24, 133 | 0.2 |
| **Age，M (SD)** |  |  |  |
| 20–44 | — | — |  |
| 45–64 | 41 | -49, 132 | 0.4 |
| ≥65 | 17 | -51, 85 | 0.6 |
| **Race/ethnicity, n (%)** |  |  |  |
| Mexican American | — | — |  |
| Other Hispanic | 61 | -90, 211 | 0.4 |
| Non-Hispanic White | -28 | -112, 56 | 0.5 |
| Non-Hispanic Black | 83 | -54, 221 | 0.2 |
| Other Race - Including Multi-Racial | 71 | -85, 228 | 0.4 |
| **Marital status, n (%)** |  |  |  |
| Married/living with partner | — | — |  |
| Never married | 29 | -80, 138 | 0.6 |
| Widowed/divorced | 15 | -73, 102 | 0.7 |
| **Education, n (%)** | |  |  |
| below high school | — | — |  |
| college or above | 37 | -48, 123 | 0.4 |
| high school | 48 | -58, 154 | 0.4 |
| **PIR, n (%)** |  |  |  |
| <1 | — | — |  |
| 1–1.99 | -153 | -274, -32 | 0.014 |
| 2–3.99 | -182 | -319, -44 | 0.01 |
| ≥4 | -218 | -349, -88 | 0.001 |
| **Smoking, n (%)** |  |  |  |
| Current smoker | — | — |  |
| Former smoker | -22 | -152, 107 | 0.7 |
| Never smoker | -39 | -163, 85 | 0.5 |
| **BMI, kg/m2, n (%)** |  |  |  |
| Low to normal (<25) | — | — |  |
| Obese (25–30) | -30 | -133, 72 | 0.6 |
| Overweight (≥30) | -29 | -123, 65 | 0.5 |
| **Hypertension, n (%)** | |  |  |
| no | — | — |  |
| yes | 110 | 49, 171 | <0.001 |
| **Heart, n (%)** |  |  |  |
| no | — | — |  |
| yes | 43 | -56, 141 | 0.4 |
| **Stroke, n (%)** |  |  |  |
| no | — | — |  |
| yes | -47 | -139, 45 | 0.3 |
| **Liver, n (%)** |  |  |  |
| no | — | — |  |
| yes | -7.3 | -150, 136 | >0.9 |
| **Cancer, n (%)** |  |  |  |
| no | — | — |  |
| yes | 19 | -76, 115 | 0.7 |
| **Hyperlipidemia, n (%)** | |  |  |
| no | — | — |  |
| yes | 103 | 23, 183 | 0.013 |
| **Respiratory, n (%)** | |  |  |
| no | — | — |  |
| yes | -0.92 | -70, 69 | >0.9 |
| **Drinke, n (%)** |  |  |  |
| Heavy drinker | — | — |  |
| Low to moderate drinker | -19 | -93, 54 | 0.6 |
| Nondrinker | 68 | -51, 187 | 0.3 |
| **OPA，Mean (SD)** | -0.79 | -2.1, 0.47 | 0.2 |
| **TPA，Mean (SD)** | -4.1 | -10, 1.8 | 0.2 |
| **LTPA，Mean (SD)** | -5.7 | -10, -0.91 | 0.02 |

*Note: ACR, Albumin/Urine Creatinine Ratio; PIR , poverty income ratio; BMI, body mass index; OPA, occupation-related physical activity; TPA, transportation-related physical activity; LTPA, leisure-time physical activity.*

# Supplementary Table S9. Subgroup analyses on the association between eGFR with TPA.

| **Subgroup Variable** | **β (95% CI)** | **P-value** | **P for interaction** |
| --- | --- | --- | --- |
| **Age，M (SD)** |  |  | 0.737 |
| 20–44 | 0.26(-0.16,0.68) | 0.225 |  |
| 45–64 | 0.28(0.01,0.56) | 0.042 |  |
| ≥65 | 0.49(-0.02,1) | 0.058 |  |
| **Sex, n (%)** |  |  | 0.037 |
| female | 1.11(0.6,1.62) | <0.001 |  |
| male | 0.5(0.21,0.79) | 0.001 |  |
| **Race/ethnicity, n (%)** |  |  | 0.443 |
| Mexican American | 0.4(-0.03,0.83) | 0.072 |  |
| Other Hispanic | 0.27(-0.2,0.75) | 0.258 |  |
| Non-Hispanic White | 0.8(0.17,1.44) | 0.013 |  |
| Non-Hispanic Black | 0.57(-0.02,1.15) | 0.057 |  |
| Other Race - Including Multi-Racial | 1.15(0.32,1.98) | 0.007 |  |
| **Education, n (%)** |  |  | 0.162 |
| below high school | 0.43(0,0.86) | 0.05 |  |
| college or above | 0.64(0.26,1.02) | 0.001 |  |
| high school | 1.12(0.53,1.7) | <0.001 |  |
| **PIR, n (%)** |  |  | 0.724 |
| <1 | 0.46(0,0.91) | 0.05 |  |
| 1–1.99 | 0.83(0.29,1.37) | 0.003 |  |
| 2–3.99 | 0.74(0.14,1.33) | 0.016 |  |
| ≥4 | 0.66(0.17,1.15) | 0.009 |  |
| **Marital status, n (%)** |  |  | 0.552 |
| Married/living with partner | 0.69(0.38,1.01) | <0.001 |  |
| Never married | 0.31(-0.29,0.92) | 0.31 |  |
| Widowed/divorced | 0.62(0.03,1.21) | 0.04 |  |
| **Smoking, n (%)** |  |  | 0.654 |
| Current smoker | 0.44(0.05,0.84) | 0.028 |  |
| Former smoker | 0.65(0.28,1.02) | 0.001 |  |
| Never smoker | 0.77(0.15,1.4) | 0.016 |  |
| **BMI, kg/m2, n (%)** |  |  | 0.065 |
| Low to normal (<25) | 0.03(-0.74,0.8) | 0.932 |  |
| Obese (25–30) | 0.95(0.58,1.32) | <0.001 |  |
| Over weight (≥30) | 0.52(0.13,0.92) | 0.01 |  |
| **Heart, n (%)** |  |  | 0.218 |
| no | 0.53(0.26,0.8) | <0.001 |  |
| yes | 0.94(0.34,1.54) | 0.002 |  |
| **Stroke, n (%)** |  |  | 0.161 |
| no | 0.59(0.33,0.84) | <0.001 |  |
| yes | 3.33(-0.69,7.35) | 0.106 |  |
| **Liver, n (%)** |  |  | 0.051 |
| no | 0.74(0.47,1.01) | <0.001 |  |
| yes | -0.18(-1.02,0.66) | 0.679 |  |
| **Cancer, n (%)** |  |  | 0.692 |
| no | 0.59(0.33,0.85) | <0.001 |  |
| yes | 0.27(-1.21,1.76) | 0.72 |  |
| **Hypertension, n (%)** |  |  | 0.341 |
| no | 0.38(0.07,0.69) | 0.016 |  |
| yes | 0.62(0.24,1) | 0.001 |  |

*Note: eGFR, estimated Glomerular Filtration Rate; PIR , poverty income ratio; BMI, body mass index; TPA, transportation-related physical activity.*

# Supplementary Table S10. Subgroup analyses on the association between eGFR with PA.

| **Subgroup Variable** | **β (95% CI)** | **P-value** | **P for interaction** |
| --- | --- | --- | --- |
| **Age，M (SD)** |  |  | 0.195 |
| 20–44 | 0.02(-0.05,0.08) | 0.639 |  |
| 45–64 | 0.08(0.03,0.13) | 0.004 |  |
| ≥65 | 0.12(0.02,0.22) | 0.019 |  |
| **Sex, n (%)** |  |  | 0.656 |
| female | 0.22(0.12,0.32) | <0.001 |  |
| male | 0.2(0.14,0.25) | <0.001 |  |
| **Race/ethnicity, n (%)** |  |  | 0.3 |
| Mexican American | 0.13(0.05,0.22) | 0.002 |  |
| Other Hispanic | 0.12(0.01,0.23) | 0.04 |  |
| Non-Hispanic White | 0.25(0.16,0.33) | <0.001 |  |
| Non-Hispanic Black | 0.19(0.09,0.29) | <0.001 |  |
| Other Race - Including Multi-Racial | 0.21(0.07,0.36) | 0.004 |  |
| **Education, n (%)** |  |  | 0.954 |
| below high school | 0.19(0.11,0.27) | <0.001 |  |
| college or above | 0.2(0.13,0.27) | <0.001 |  |
| high school | 0.21(0.11,0.3) | <0.001 |  |
| **PIR, n (%)** |  |  | 0.948 |
| <1 | 0.2(0.11,0.28) | <0.001 |  |
| 1–1.99 | 0.21(0.12,0.3) | <0.001 |  |
| 2–3.99 | 0.18(0.09,0.27) | <0.001 |  |
| ≥4 | 0.18(0.08,0.28) | 0.001 |  |
| **Marital status, n (%)** |  |  | 0.527 |
| Married/living with partner | 0.17(0.11,0.22) | <0.001 |  |
| Never married | 0.16(0.05,0.28) | 0.007 |  |
| Widowed/divorced | 0.23(0.12,0.34) | <0.001 |  |
| **Smoking, n (%)** |  |  | 0.339 |
| Current smoker | 0.13(0.06,0.2) | <0.001 |  |
| Former smoker | 0.2(0.13,0.27) | <0.001 |  |
| Never smoker | 0.22(0.13,0.31) | <0.001 |  |
| **BMI, kg/m2, n (%)** |  |  | 0.608 |
| Low to normal (<25) | 0.21(0.08,0.34) | 0.001 |  |
| Obese (25–30) | 0.22(0.15,0.28) | <0.001 |  |
| Over weight (≥30) | 0.16(0.09,0.24) | <0.001 |  |
| **Heart, n (%)** |  |  | 0.153 |
| no | 0.16(0.12,0.21) | <0.001 |  |
| yes | 0.27(0.13,0.41) | <0.001 |  |
| **Stroke, n (%)** |  |  | 0.052 |
| no | 0.18(0.13,0.22) | <0.001 |  |
| yes | 0.47(0.17,0.76) | 0.003 |  |
| **Liver, n (%)** |  |  | 0.873 |
| no | 0.2(0.15,0.24) | <0.001 |  |
| yes | 0.18(0.04,0.33) | 0.013 |  |
| **Cancer, n (%)** |  |  | 0.024 |
| no | 0.17(0.12,0.22) | <0.001 |  |
| yes | 0.4(0.22,0.59) | <0.001 |  |
| **Hypertension, n (%)** |  |  | 0.107 |
| no | 0.13(0.07,0.2) | <0.001 |  |
| yes | 0.21(0.15,0.27) | <0.001 |  |

*Note: eGFR, estimated Glomerular Filtration Rate; PIR , poverty income ratio; BMI, body mass index; PA, total physical activity.*

# Supplementary Table S11. Subgroup analyses on the association between ACR with LTPA.

| **Subgroup Variable** | **β (95% CI)** | **P-value** | **P for interaction** |
| --- | --- | --- | --- |
| **Age，M (SD)** |  |  | 0.214 |
| 20–44 | 5.16(-13.63,23.95) | 0.59 |  |
| 45–64 | -9.66(-21.29,1.96) | 0.103 |  |
| ≥65 | -10.07(-20.23,0.08) | 0.052 |  |
| **Sex, n (%)** |  |  | 0.246 |
| female | -13.36(-28.83,2.12) | 0.091 |  |
| male | -3.49(-11.21,4.23) | 0.376 |  |
| **Race/ethnicity, n (%)** |  |  | 0.291 |
| Mexican American | -12.25(-25.91,1.4) | 0.079 |  |
| Other Hispanic | 14.91(-12.97,42.79) | 0.295 |  |
| Non-Hispanic White | -7.42(-17.49,2.64) | 0.149 |  |
| Non-Hispanic Black | -7.59(-25.05,9.86) | 0.394 |  |
| Other Race - Including Multi-Racial | -10.65(-26.75,5.45) | 0.196 |  |
| **Education, n (%)** |  |  | 0.212 |
| below high school | -13.96(-31.8,3.88) | 0.125 |  |
| college or above | -6.55(-15.21,2.1) | 0.138 |  |
| high school | 6.5(-10.29,23.29) | 0.448 |  |
| **PIR, n (%)** |  |  | 0.781 |
| <1 | -2.26(-25.06,20.53) | 0.846 |  |
| 1–1.99 | -10.84(-26.87,5.2) | 0.186 |  |
| 2–3.99 | -5.48(-16.72,5.75) | 0.339 |  |
| ≥4 | -1.01(-5.79,3.78) | 0.68 |  |
| **Marital status, n (%)** |  |  | 0.261 |
| Married/living with partner | -1.42(-9.72,6.88) | 0.737 |  |
| Never married | -12.05(-31.05,6.96) | 0.215 |  |
| Widowed/divorced | -15.42(-34.25,3.4) | 0.109 |  |
| **Smoking, n (%)** |  |  | 0.733 |
| Current smoker | -6.2(-22.19,9.78) | 0.447 |  |
| Former smoker | -8.56(-19.26,2.14) | 0.117 |  |
| Never smoker | -2.27(-14.09,9.55) | 0.707 |  |
| **BMI, kg/m2, n (%)** |  |  | 0.816 |
| Low to normal (<25) | -9.29(-26.02,7.44) | 0.277 |  |
| Obese (25–30) | -7.17(-16.83,2.5) | 0.146 |  |
| Over weight (≥30) | -3.01(-16.5,10.48) | 0.662 |  |
| **Heart, n (%)** |  |  | 0.094 |
| no | -2.74(-10.44,4.96) | 0.486 |  |
| yes | -18.21(-36.24,-0.18) | 0.048 |  |
| **Stroke, n (%)** |  |  | 0.498 |
| no | -5.34(-12.73,2.05) | 0.157 |  |
| yes | -20.56(-51.56,10.44) | 0.195 |  |
| **Liver, n (%)** |  |  | 0.434 |
| no | -4.9(-12.39,2.59) | 0.2 |  |
| yes | -13.5(-38.03,11.04) | 0.282 |  |
| **Cancer, n (%)** |  |  | 0.488 |
| no | -5.07(-12.34,2.2) | 0.172 |  |
| yes | -13.9(-41.59,13.79) | 0.326 |  |
| **Hypertension, n (%)** |  |  | 0.885 |
| no | -4.29(-9.77,1.18) | 0.124 |  |
| yes | -5.35(-17.16,6.46) | 0.375 |  |

*Note: ACR, Albumin/Urine Creatinine Ratio; PIR , poverty income ratio; BMI, body mass index; LTPA, leisure-time physical activity.*
